# Rare Variants of the *SMN1* Gene Detected during Neonatal Screening

**DOI:** 10.3390/genes15070956

**Published:** 2024-07-21

**Authors:** Maria Akhkiamova, Aleksander Polyakov, Andrey Marakhonov, Sergey Voronin, Elena Saifullina, Zulfiia Vafina, Kristina Michalchuk, Svetlana Braslavskaya, Alena Chukhrova, Nina Ryadninskaya, Sergey Kutsev, Olga Shchagina

**Affiliations:** 1 Research Centre for Medical Genetics, Moskvorechie Str., 1, 115522 Moscow, Russia; apol@dnalab.ru (A.P.); marakhonov@generesearch.ru (A.M.); voronin.sv@med-gen.ru (S.V.); grstina@yandex.ru (K.M.); braslav_dna@mail.ru (S.B.); achukhrova@yandex.ru (A.C.); outremal@yandex.ru (N.R.); kutsev@mail.ru (S.K.); schagina@dnalab.ru (O.S.); 2 Bashkir State Medical University, Lenin Str., 3, 450008 Ufa, Russia; riledin@mail.ru; 3 Republic of Tatarstan Ministry of Healthcare Autonomous Public Healthcare, Institution Republic Clinical Hospital, Orenburgskiy Tract Str., 138, 420064 Kazan, Russia; vzulfia@mail.ru

**Keywords:** spinal muscular atrophy, 5q SMA, NBS, VUS, splicing

## Abstract

During the expanded neonatal screening program conducted in 2023, we analyzed samples obtained from 1,227,130 out of 1,256,187 newborns in the Russian Federation in order to detect 5q spinal muscular atrophy (5q SMA). Within the 253-sample risk group formed based on the results of the first screening stage, 5 samples showed a discrepancy between the examination results obtained via various screening methods and quantitative MLPA (used as reference). The discrepancy between the results was caused by the presence of either a c.835-18C>T intronic variant or a c.842G>C p.(Arg281Thr) missense variant in the *SMN1* gene, both of which are located in the region complementary to the sequences of annealing probes for ligation and real-time PCR. Three newborns had the c.835-18C>T variant in a compound heterozygous state with a deletion of exons 7–8 of the *SMN1* gene, one newborn with two copies of the *SMN1* gene had the same variant in a heterozygous state, and one newborn had both variants—c.835-18C>T and c.842G>C p.(Arg281Thr)—in a compound heterozygous state. Additional examination was carried out for these variants, involving segregation analysis in families, carriage analysis in population cohorts, and RNA analysis. Based on the obtained results, according to the ACMG criteria, the c.835-18C>T intronic variant should be classified as likely benign, and the c.842G>C p.(Arg281Thr) missense substitution as a variant of uncertain clinical significance. All five probands are under dynamic monitoring. No 5q SMA symptoms were detected in these newborns neonatally or during a 1-year follow-up period.

## 1. Introduction

Proximal spinal muscular atrophy 5q (5q SMA) is a severe autosomal recessive neuromuscular disorder, characterized by progressive symptoms of flaccid paralysis and muscular atrophy because of the degeneration of α-motor neurons in the anterior horn of the spinal cord and the lower brainstem [1].

Proximal 5q SMA is caused by pathogenic variants in the survival of motor neuron gene (SMN1). Homozygous deletions of exons 7 and 8 in this gene are the cause of SMA in 94–96% of all cases [2]. In 3–6% of all SMA cases, the disease is caused by a compound heterozygous state of deletions and minor mutations (missense, nonsense, or small deletions/insertions) [3]. There have been studies describing singular cases of SMA caused by biallelic minor variants [4].

The SMN1 gene (OMIM:*600354) is mapped on chromosome 5 in locus 5q12.2-q13.3 and has a centromeric copy—the survival of motor neuron gene 2 (SMN2, OMIM:*601627). Both genes include nine exons (1, 2a, 2b, 3–8) and have a five-nucleotide discrepancy (three in introns 6 and 7, two in exons 7 and 8). The c.840C>T replacement in exon 7 of SMN2 creates a binding site for a splicing repressor and causes the differences in gene transcripts. The SMN protein consists of 294 amino acids, has a molecular mass of 38 kD, and functions in the nucleus and cytoplasm. Full-length SMN (FL-SMN) is localized in the nucleus within subnuclear structures called gems, which are associated with Cajal bodies. FL-SMN is a product of the SMN1 gene. SMN2 produces 90% of all truncated protein and 10% of all full-length functioning protein. In the majority of cases, the SMN2 transcript does not contain ex7 (SMNΔ7); as a result, the expressed protein is functionally impaired, and the ubiquitin proteosome system leads to its degradation. A decrease in SMN levels causes the 5q SMA phenotype [5].

In 2023, the Russian Federation introduced expanded neonatal screening (NBS) for 36 disorders, including 5q spinal muscular atrophy (5q SMA) [6]. The 5q SMA screening process was carried out in ten federal neonatal screening centers via real-time PCR using the following kits: TK-SMA (Generium, Moscow, Russia) and NeoScreen SMA/TREC/KREC (DNA-Technology, Moscow, Russia).

The whole blood samples showing positive or uncertain results were referred to the Research Centre for Medical Genetics (RCMG), where they underwent second-tier real-time PCR and quantitative MLPA for the genetic confirmation of the diagnosis and subsequent assignment of treatment.

In 2023, 1,256,187 children were born in the Russian Federation and 1,227,130 were tested for 5q SMA, with consent obtained from their mothers [6]. The 5q SMA risk group consisted of 253 referred newborns. In total, 121 of them had the exon 7 deletion in the *SMN1* gene in a homozygous state. Five samples showed discordant results when analyzed with various screening methods and the reference quantitative MLPA. The aim of the current study is to establish the molecular causes of this discrepancy, as well as the pathogenicity of detected variants.

## 2. Materials and Methods

The materials used for this study were DNA samples from five newborns, as well as thirteen of their family members (parents and siblings). The population control cohort included DNA samples from Bashkir, Tatar, Chuvash, and Mari people from the archive of the DNA diagnostics laboratory at the RCMG. Aside from that, we examined RNA samples from the newborn with the c.835-18C>T variant in a compound heterozygous state with a deletion of exons 7 and 8 of the *SMN1* gene, and from the newborn with compound heterozygous variants c.835-18C>T and c.842G>C p.(Arg281Thr), as well as the latter’s family members. Control RNA samples were extracted from the blood of four healthy Russian citizens with a known number of copies of *SMN1* and *SMN2*.

DNA was extracted using the Wizard Genomic DNA Purification Kit (Promega, Madison, WI, USA) according to the manufacturer’s protocol. RNA was extracted using the ExtractRNA reagent kit (Evrogen, Moscow, Russia), and reverse transcription was carried out using the MMLV RT kit (Evrogen, Moscow, Russia) according to the manufacturer’s protocols. The homozygous exon 7 deletion in the *SMN1* gene was detected during screening using the SALSA MC002 SMA Newborn Screen kit (MRC Holland, Amsterdam, The Netherlands), TK-SMA (Generium, Moscow, Russia), nescreen SMA/TREC/KREC (DNA Technology, Moscow, Russia), and the Eonic SCID-SMA kit (Wallac Oy, Turku, Finland), as previously described and according to the manufacturers’ protocols. The number of exon 7 and 8 copies in the *SMN1* and *SMN2* genes was determined using the SALSA MLPA Probemix P060 SMA kit (MRC Holland, Amsterdam, The Netherlands) according to the manufacturer’s protocol. We also used the PCR-restriction fragment length polymorphism (RFLP) method to detect the deletion of exons 7 and 8 in the *SMN1* and *SMN2* genes [7]. The nucleotide sequence of the *SMN1* gene was analyzed via direct automated Sanger sequencing with an ABI PRISM 3500 genetic analyzer (Thermo Fisher Scientific, Waltham, MA, USA) using the manufacturer’s reagents and protocols. We used the previously published primer sequences and PCR conditions [8]. To determine the c.835-18C>T variant and its location in the *SMN1* gene, we developed a method of allele-specific ligase reaction using the following probes: MSMN 835-18FC GTTCGTACGTGAATCGCGGTACATAGCTATCTATGTCTATATAGCTATTTTTTTTAACTTC, MSMN 835-18FT GTTCGTACGTGAATCGCGGTACGCTATCTATGTCTATATAGCTATTTTTTTTACTTT, MSMN 835-18LINKER CTTTATTTTCCTTACAGGGTTTC, MSMN 835-18R AGACAAAATCAAAAAGAAGGAAGGGATGCGATCCGATGCCTTCATG. These probes are simultaneously specific to the c.835-18C>T variant and the point of difference between *SMN1* and *SMN2*, which allows us to establish the exact location of the sequence variant. cDNA was analyzed using PCR with the following primer sequences: SMN3/4F GTTCGTACGTGAATCGCGGTACGCTCAAGAGAATGAAAATGAAAGC, SMN7/8R GCCAGCATTTCTCCTTAATTTAAGG, which are complementary to the junctions between exons 3–4 and 7–8 of the SMN1 gene, with an additional sequence complementary to the fluorescence-labeled UNIQV GTTCGTACGTGAATCGCGGTAC primer, which allows us to detect the results by fragment analysis with an ABI PRISM 3500 (Thermo Fisher Scientific, USA).

## 3. Results

### 3.1. Discrepant Results of Analysis

As a result of the examination of the 5q SMA risk group formed during NBS in the reference center, five samples were shown to produce discrepant results during analysis with various screening methods and the reference method of quantitative MLPA.

#### 3.1.1. New Variations

**Figure 1:**
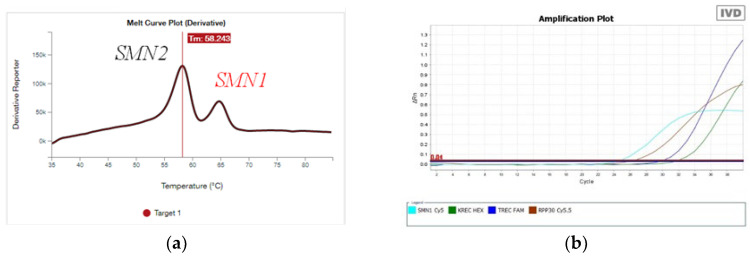

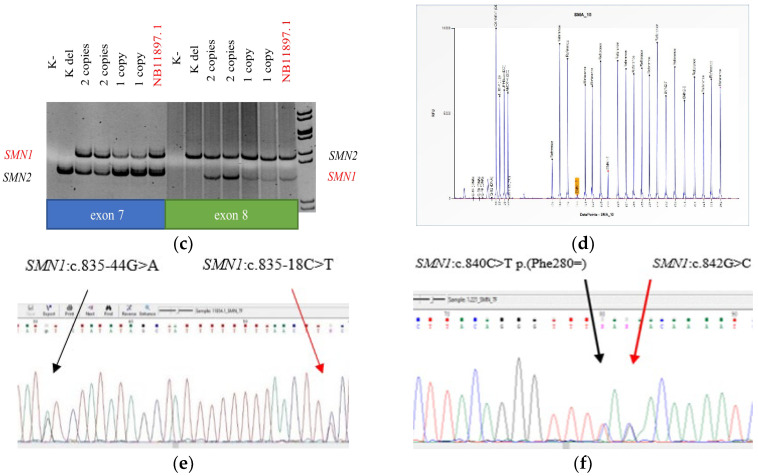
(**a**) The high-resolution melting curve analysis of the SMN1 and SMN2 genes. Amplifications of the SMN2 gene melt at a temperature of 57 °C; SMN1, 65 °C. (**b**) Real-time PCR. The blue line corresponds to the amplification of the locus of the SMN1 gene. (**c**) PCR-RFLP analysis, featuring the presence of exon 7 of the SMN1 gene. (**d**) Quantitative MLPA, featuring homozygous deletion of exon 7 of the SMN1 gene, exon 8 of the SMN1 gene *n* = 1 copy, exon 7 of the SMN2 gene *n* = 2 copies, and exon 8 of the SMN2 gene *n* = 2 copies. (**e**) Sanger sequencing, featuring heterozygous substitution c.835-18C>T in intron 6 of the SMN1 gene (NM_000344.4). (**f**) Sanger sequencing, featuring heterozygous substitution of p.842G>C p.(Arg281Thr) in exon 7 of the SMN1 gene (NM_000344.4).

All five samples were examined using high-definition melting curve analysis (Figure 1a), during which we found a peak that corresponds to exon 7 of the SMN1 gene at 65 °C. In addition, real-time PCR (Figure 1b) showed a light blue peak corresponding to exon 7 of the SMN1 gene. In all five samples, PCR-RFLP analysis (Figure 1c) revealed the presence of exon 7 of the SMN1 gene. However, quantitative analysis of MLPA (Figure 1d) showed homozygous deletion of exon 7 of the SMN1 gene, with an equal number of copies of exons 7 and 8 of the SMN2 gene in all newborns. Thus, we used MLPA to identify an atypical chimeric genotype that raised our concerns. Sanger sequencing (Figure 1e,f) for the probands’ parents and siblings detected the c.835-18C>T and c.842G>C p.(Arg281Thr) variants in the SMN1 gene in the sequence complementary to the region of quantitative MLPA probe hybridization.

#### 3.1.2. Novel Detection System

**Figure 2:**
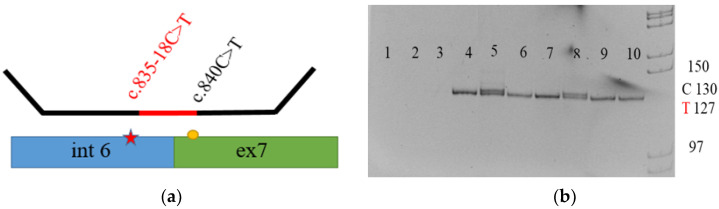
(**a**) The scheme of allele-specific MLPA. The location of the variant c.835-18C>T in the SMN1 gene is determined by using a primer at the point of difference (c.840C>T) between the SMN1 and SMN2 genes. (**b**) Allele-specific MLPA. Lane 1 is a negative control sample; lanes 2 and 3 represent the absence of amplification in samples with a homozygous deletion in the SMN1 gene; lane 4 is a control sample, with two copies of the SMN1 gene with c.835-18C= in a homozygous state; lanes 5 and 8 are samples with two copies of the SMN1 gene with a heterozygous c.835-18C>T substitution; lanes 6, 7, 9, and 10 are samples with one copy of the SMN1 gene with a hemizygous c.835-18C>T substitution. Fragment lengths corresponding to the presence or absence of the c.835-18C>T variant are also presented.

To detect the c.835-18C>T variant, we developed a system of allele-specific MLPA, which allows us to establish the presence of the variant, as well as its location in either the SMN1 or SMN2 gene. DNA samples from all five newborns, their parents, and their siblings were analyzed using allele-specific MLPA. The examination of the parents showed that in three cases the c.835-18C>T variant was inherited from one of the parents, while the second parent was found to carry the SMN1 deletion (Figure 2). The mother of one of the probands had the c.835-18C>T variant in a hemizygous state (Table 1).

### 3.2. Segregation Analysis

**Figure 3:**
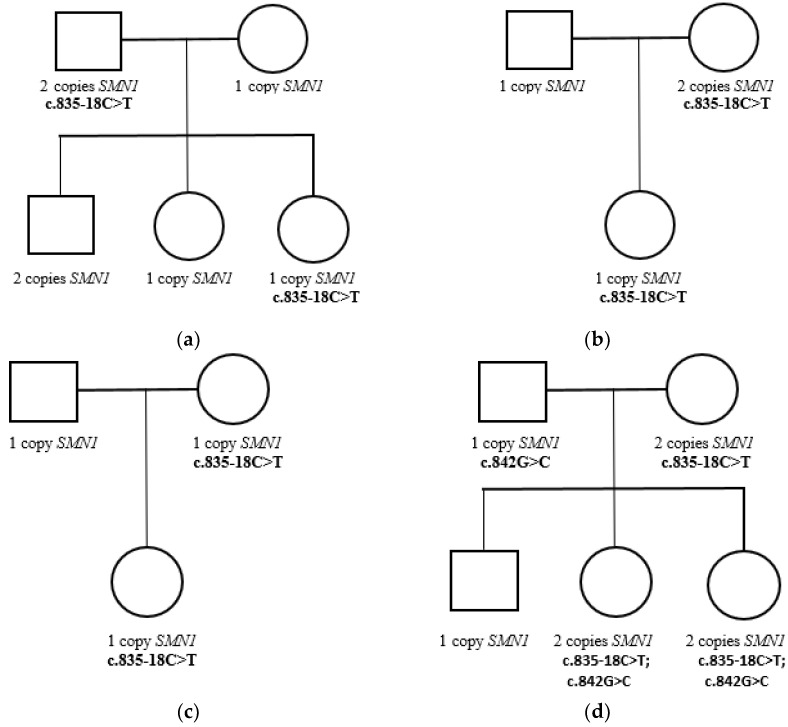
(**a**) The pedigree of the NB11897.1 newborn’s family. (**b**) The pedigree of the NB11907.1 newborn’s family. (**c**) The pedigree of the NB11920.1 newborn’s family. (**d**) The pedigree of the NB11940.1 newborn’s family.

Allele-specific MLPA and Sanger sequencing of the parents and siblings of one newborn detected the c.835-18C>T and c.842G>C p.(Arg281Thr) variants in the SMN1 gene. The variants were detected in a compound heterozygous state in the proband and his sister, the c.835-18C>T variant was detected in his mother in a heterozygous state, and the c.842G>C variant was detected in his father in a hemizygous state (Table 1, Figure 3). The c.842G>C variant was also detected in a newborn from the Republic of Dagestan with two copies of the SMN1 gene, but segregation analysis was not carried out for that family (Table 1).

### 3.3. cDNA Amplification Results

**Figure 4:**
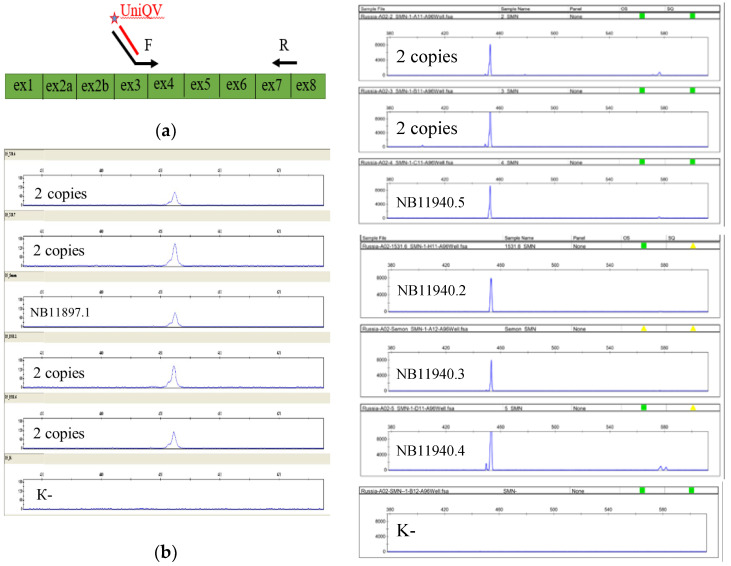
(**a**) The layouts of the primers. The forward, with an additional sequence complementary to the universal labeled primer, is complementary to the sequence of the end of exon 3 and the beginning of exon 4 of the *SMN1* gene; the reverse is complementary to the reversed sequence of the end of exon 7 and the beginning of exon 8 of the *SMN1* gene. (**b**) Fragment analysis of the newborns’ cDNA. There were no differences between the examined samples and the control ones in terms of the number and length of fragments, and there was a certain non-specificity.

We next assessed the possible influence of the novel variants on the splicing of transcript isoform NM_000344.4 of the *SMN1* gene. The amplification of cDNA (Figure 4) obtained from the leukocytes of newborns with the c.835-18C>T and c.842G>C variants using RNA-specific primers did not indicate any difference between the examined samples and the control samples in terms of the number and length of fragments. The control samples did not have previously described variants and underwent copy number analysis, which detected two and three copies of the *SMN1* gene.

### 3.4. Analysis of Ethnic Backgrounds of Newborns with Novel Variants

The blood samples of newborns with novel variants were received from the Republics of Bashkortostan and Tatarstan. Ethnically, the parents of newborn NB11897.1 were Chuvash; NB11907.1, Mari; NB11920.1, Russian and Tatar; NB11940.1, Tatar. The retrospective analysis of data using quantitative MLPA did not point to the presence of the c.835-18C>T variant (because of discordant signals from the SMN1/2 locus—the ex7 genotype, with 0 copies of the *SMN1* gene; or the ex8 genotype, with 1 or 2 copies of the *SMN1* gene along with an equal number of copies of exons 7 and 8 of the *SMN2* gene) in 28 DNA samples from patients with symptoms and 0 copies of *SMN1* from these Republics. Examination of a mixed cohort of 285 people from the Republics of Bashkortostan, Tatarstan, Chuvashia, and Mari El revealed the c.835-18C>T variant in one person, where it was in a heterozygous state. All five revealed newborns are currently under ambulatory monitoring; the probands, aged 15, 12, 10, 8, and 7 months, do not show any symptoms of 5q SMA to date.

## 4. Discussion

During the neonatal screening, we detected the c.835-18C>T and c.842G>C variants in the *SMN1* gene. The c.835-18C>T (rs1554082373) intronic variant has been registered in the ClinVar database (ID: 495823) as a variant of uncertain clinical significance (VUS); it has not been found in the gnomAD v2.1.1 or RuSeq databases, and its frequency in the RuExac database is 0.0172% [9,10,11,12]. The missense variant c.842G>C p.(Arg281Thr) (rs1561503124) has been described in ClinVar (ID: 632986) as a VUS, found in gnomAD with a frequency of 0.0007% and not described in the RuSeq or RuExac databases [11,12,13,14]. According to the prediction programs NetGene2, Softberry SPLM, and BDGP Splice Site Prediction, the c.835-18C>T variant does not affect the conservative splice site and does not lead to the formation of any new splice sites. According to MutationTaster, PolyPhen-2, and MetaRNN, the c.842G>C variant does not affect the structure or function of proteins. During the examination of a cohort consisting of 285 Chuvash, Mari, and Tatar people, the c.835-18C>T variant was detected in a heterozygous state in 1 person; no ethnic accumulation was noted. The amplification of cDNA extracted from the leukocytes of newborns with the c.835-18C>T and c.842G>C variants using RNA-specific primers did not present any difference between the examined samples and the control samples in terms of the length and number of fragments. The c.835-18C>T variant was detected in the proband’s mother in a heterozygous state, while the c.842G>C variant was detected in the proband’s father in a hemizygous state, which allows us to assume that the variants are benign. The newborns, aged 15, 12, 10, 8, and 7 months, show no symptoms of 5q SMA. According to ACMG recommendations, the c.835-18C>T intronic variant fits the criteria of BS3, BP4, and the c.842G>C p.(Arg281Thr) missense variant, BS3, PM2, BP4. These criteria indicate that the c.835-18C>T intronic variant should be classified as likely benign, and the c.842G>C p.(Arg281Thr) missense variant in the *SMN1* gene should be classified as VUS. The c.842G>C variant has been detected once, in a newborn from the United States with an uncertain screening result during additional testing [15]. The researchers carried out an array of tests and established that the c.842G>C variant does not impair the oligomerization of the YG box or the function of the SMN1 product [16]. The screening for 5q SMA in the United Kingdom has detected the following variants: c.5C>G p.(Ala2Gly)—likely pathogenic—c.97_99delATTinsTGA p.(Ile33*), c.419A>T p.(Asp140Val), c.475-2A>T, c.815A>G p.(Tyr272Cys), c.821C>T p.(Thr274lle), and c.835-18_835-12del. According to the ACMG criteria, the authors evaluated the variants to be likely pathogenic or VUS [17]. Data presented in the literature describe variants located in the same regions as the examined ones: c.835-13A>G [10,18], c.835-14T>C [10], c.835-19T>A [10,19], c.835-21C>T [10], c.835-24T>A [10,20], c.842G>A p.(Arg281Lys) [10], and c.842G>T p.(Arg281Ile) [10]. According to gnomAD and ClinVar, these variants should be interpreted as VUS; other databases and articles do not describe these variants. These accidental findings from the neonatal screening for 5q SMA raise multiple concerns among researchers regarding the interpretation, monitoring, and treatment of the variants. However, we should evaluate the variants’ pathogenicity very cautiously due to the absence of clinical symptoms in the newborns.

## 5. Conclusions

Thus, according to the ACMG criteria, the c.835-18C>T intronic variant should be classified as likely benign, while the c.842G>C p.(Arg281Thr) missense variant in the *SMN1* gene should be classified as VUS. During NBS, molecular genetic methods may show accidental findings, which require precise clinical interpretation due to the absence of symptoms, as well as additional testing to establish the variant pathogenicity. These findings were detected in 4% of Russian newborns from the 5q SMA risk group formed during the initial screening analysis.

Multiple *SMN1* variants have been described as affecting protein function; however, the interpretation of previously undescribed variants accidentally detected during neonatal screening may be complicated by the absence of clinical symptoms.

## Figures and Tables

**Table 1:** Genotypes of newborns, parents, and siblings.

Tested	*SMN1* (ex7, ex8)	*SMN2* (ex7, ex8)	Sequencing (NM_000344.4)
Proband NB11897.1	1 copy	2 copies	c.[835-18C>T];[0]
Father NB11897.2	2 copies	2 copies	c.[835-18C>T];[=]
Mother NM11897.3	1 copy	2 copies	Not detected
Brother NB11897.4	2 copies	2 copies	Not detected
Sister NB11897.5	1 copy	2 copies	Not detected
Proband NB11907.1	1 copy	3 copies	c.[835-18C>T];[0]
Father NB11907.2	1 copy	3 copies	Not detected
Mother NB11907.3	2 copies	2 copies	c.[835-18C>T];[=]
Proband NB11920.1	1 copy	2 copies	c.[835-18C>T];[0]
Father NB11920.2	1 copy	2 copies	Not detected
Mother NB11920.3	1 copy	2 copies	c.[835-18C>T];[0]
Proband NB11940.1	2 copies	2 copies	c.[835-18C>T];[842G>C]
Father NB11940.2	1 copy	3 copies	c.[842G>C];[0]
Mother NB11940.3	2 copies	2 copies	c.[835-18C>T];[=]
Brother NB11940.4	1 copy	3 copies	Not detected
Sister NB11940.5	2 copies	2 copies	c.[835-18C>T];[842G>C]
Proband NB1.221	1 copy	2 copies	c.[842G>C];[=]

## Data Availability

The original contributions presented in the study are included in the article; further inquiries can be directed to the corresponding author.
